# Improving management of comorbidity in patients with colorectal cancer using comprehensive medical assessment: a pilot study

**DOI:** 10.1186/s12885-020-6526-z

**Published:** 2020-01-20

**Authors:** Virginia Signal, Christopher Jackson, Louise Signal, Claire Hardie, Kirsten Holst, Marie McLaughlin, Courtney Steele, Diana Sarfati

**Affiliations:** 1 Department of Public Health, University of Otago, PO Box 7343, South, Wellington, 6242 New Zealand; 2 Department of Medicine, University of Otago, Dunedin: Southern Blood and Cancer Service, Southern District Health Board, Dunedin, New Zealand; 3 School of Medicine and Health Sciences at Palmerston North, University of Otago, Wellington: Cancer Screening Treatment and Support, MidCentral District Health Board, Palmerston North, New Zealand; 4 Elder Health, MidCentral District Health Board, Palmerston North, New Zealand; 5 Department of Medicine, University of Otago, Dunedin: Older Persons Health, Southern District Health Board, Dunedin, New Zealand

**Keywords:** Neoplasms, Comorbidity, Health services, Quality improvement, Interventions, Chemotherapy tolerance

## Abstract

**Background:**

Screening for and active management of comorbidity soon after cancer diagnosis shows promise in altering cancer treatment and outcomes for comorbid patients. Prior to a large multi-centre study, piloting of the intervention (comprehensive medical assessment) was undertaken to investigate the feasibility of the comorbidity screening tools and proposed outcome measures, and the feasibility, acceptability and potential effect of the intervention.

**Methods:**

In this pilot intervention study, 72 patients of all ages (36 observation/36 intervention) with newly diagnosed or recently relapsed colorectal adenocarcinoma were enrolled and underwent comorbidity screening and risk stratification. Intervention patients meeting pre-specified comorbidity criteria were referred for intervention, a comprehensive medical assessment carried out by geriatricians. Each intervention was individually tailored but included assessment and management of comorbidity, polypharmacy, mental health particularly depression, functional status and psychosocial issues. Recruitment and referral to intervention were tracked, verbal and written feedback were gathered from staff, and semi-structured telephone interviews were conducted with 13 patients to assess screening tool and intervention feasibility and acceptability. Interviews were transcribed and analysed thematically. Patients were followed for 6–12 months after recruitment to assess feasibility of proposed outcome measures (chemotherapy uptake and completion rates, grade 3–5 treatment toxicity, attendance at hospital emergency clinic, and unplanned hospitalisations) and descriptive data on outcomes collated.

**Results:**

Of the 29 intervention patients eligible for the intervention, 21 received it with feedback indicating that the intervention was acceptable. Those in the intervention group were less likely to be on 3+ medications, to have been admitted to hospital in previous 12 months, or to have limitations in daily activities. Collection of data to measure proposed outcomes was feasible with 55% (6/11) of intervention patients completing chemotherapy as planned compared to none (of 14) of the control group. No differences were seen in other outcome measures. Overall the study was feasible with modification, but the intervention was difficult to integrate into clinical pathways.

**C**onclusions**:**

This study generated valuable results that will be used to guide modification of the study and its approaches prior to progressing to a larger-scale study.

**Trial registration:**

Retrospective, 26 August 2019, ACTRN12619001192178.

## Background

Comorbidity is the occurrence of other diseases alongside the disease of interest and is common among patients with cancer [1–4]. At least one comorbid condition is recorded in approximately half of all patients with cancer, and two or more conditions are present in a third of patients [5, 6]. This is partly because both cancer and comorbid conditions increase in incidence with ageing, and because cancer and some comorbid conditions share common risk factors [7]. The presence of comorbidity has significant implications for cancer treatment decisions and outcomes [1, 3, 8–12]. Comorbidity substantially reduces the likelihood that an individual will receive cancer treatment [3, 8, 13–16], and adversely impacts cancer survival [1, 8, 10, 17, 18]. However, this adverse impact can be moderated through receipt of definitive treatment [10, 19–22], Despite the importance of comorbidity, comorbid cancer patients are often excluded from clinical trials [7, 8] and most treatment guidelines focus on the management of one disease only [9]. Thus, there is little evidence relating to cancer treatment that includes comorbid patients.

Comprehensive geriatric assessments (CGA) are increasingly used to assess patient functional and nutritional status, psychological and social wellbeing, presence of geriatric syndromes and polypharmacy, and to better manage these factors in the geriatric cancer patient [23–28]. While few studies have assessed the extent to which CGA or similar broad-based medical review at the time of cancer diagnosis actually alter cancer outcomes, those that have, without exception, found that such interventions have a positive impact [29–32]. However, comorbidity can occur independent of ageing and can impact on treatment or outcome independently [7]. To date, no studies have determined the value of such an intervention for cancer patients of all ages – a comprehensive medical assessment (CMA) - nor have any studies reported qualitative data pertaining to such an approach.

New Zealand (NZ) has a publicly funded national health system with six cancer treatment centres delivering the majority of cancer treatment to its five million population. Prior to embarking on a large multi-centre intervention study we wished to address several questions regarding feasibility. Therefore, this study assessed the feasibility of the comorbidity screening tools and of collecting key data including proposed outcome measures, the acceptability and potential effect of the CMA, and the feasibility of a larger study.

## Methods

### Study design

This non-randomised pilot intervention study compared geriatrician-delivered CMA with usual oncology care. The study was followed by patient and key informant interviews. It was conducted in three hospitals within two cancer treatment centres in NZ between May 2016 and November 2017. Ethical approval was given by the Southern Health and Disability Ethics Committee, New Zealand (15/STH/193 and 15/STH/193/AM07). Although a non-randomised design was used, where possible the study adheres to CONSORT guidelines (See Additional file 1: CONSORT extension for pilot and feasibility trials checklist).

### Patient eligibility and identification

Patients were included if they had newly diagnosed or recently relapsed colorectal adenocarcinoma. Patients were excluded if they had locally recurrent rectal cancer, were treated solely with polypectomy, or had a life expectancy of less than 3 months. There were no restrictions based on age, performance status, or involvement in clinical trials. Potential participants were identified at multi-disciplinary meetings (MDM), by colorectal cancer nurses, or at oncology clinics, and referred into the study.

### Study processes and intervention

This study was conducted in two phases: an observation phase (May–October 2016), and an intervention phase (October 2016–May 2017). In both phases patients were recruited to complete a baseline self-reported questionnaire on their current health status. This comorbidity screening tool included questions on long-term conditions (New Zealand Health Survey [33]), functional and social status, previous health service use, medications (CGA-GOLD [29]), quality of life (EORTC-QLQ-C30 [34]), and a screen for depression [35, 36]. Research nurses also completed a screen for comorbidity through medical note review and undertook a check for medication interactions [37]. Patient eligibility for referral to CMA was based on these data. (See Additional file 2: CMA Criteria).

The CMA referral criteria included having a chronic condition that adversely affected health status, function, or quality of life; being on three or more regular medications; medication interactions at levels C (monitor therapy), D (consider therapy modification) or X (avoid combination); or hospitalisation within the preceding 12 months (other than for cancer treatment). The individual CMA criteria were stratified according to a traffic light system with high-risk factors graded as red and medium-risk factors as yellow. Patients met eligibility for CMA by either meeting any one red criterion or any two yellow criteria.

In the observation phase, all patients received usual care after the relevant data were collected. In the intervention phase, patients who were eligible for CMA were referred to study specific geriatrician/s within outpatient clinics. The intention was for CMAs to be delivered soon after diagnosis. Each CMA was tailored to the individual patient but included assessment and management of comorbidity, polypharmacy, mental health with a particular focus on depression, functional status and psychosocial issues. The CMA and resultant interventions were communicated to the oncology team and the patient’s primary care practitioner.

### Evaluation and analysis methods

Recruitment overall, and eligibility for and receipt of CMA were tracked. All patients were followed for 6–12 months after recruitment to assess the potential effect of the CMA and feasibility of proposed outcome measures. The impact of CMA on chemotherapy tolerance and toxicity was determined by comparing the intervention and observation patients in terms of rates of chemotherapy uptake, completion of chemotherapy as planned (defined as completing initially planned chemotherapy course without later modification or early discontinuation), grade 3–5 toxicity (CTCAE v4.03 [38]), attendance at hospital emergency clinic, and unplanned hospitalisations. As this was a pilot study, some data were collected to assess the feasibility of collecting them within a larger study and no formal hypothesis testing was planned,thus descriptive statistics only are reported.

On completion of this study, a third phase was undertaken using qualitative methods (June – November 2017). Semi-structured telephone interviews were held with 13 patients, including six patients who received a CMA. Questions regarding participation in the study overall and on living with cancer and comorbidity were asked of all patients. Those who had a CMA were also asked about their satisfaction with the CMA and how the CMA and its processes could be altered in the future. All interviews were conducted by VS, digitally recorded and transcribed. The transcripts were analysed thematically by VS and CS with iterative discussion of emerging and final themes and input from all researchers. To additionally assess study feasibility, interviews were held and written feedback gathered from the two geriatricians who conducted the majority of the CMA, and study findings were workshopped at national cancer nurse coordinator meetings.

## Results

### Patient characteristics and levels of comorbidity

72 patients consented to participate; 36 in each of the observation and intervention groups. A number of differences were seen in patient and disease-related characteristics between observation and intervention phase patients, with intervention patients slightly younger on average and more likely to female, diagnosed with earlier stage disease and to have had surgery for their primary tumour (Table 1).

**Table 1:** Patient and disease factors (total cohort)

	Observation		Intervention		Total	
	#	%	#	%	#	%
Age						
Mean	74	*–*	73	*–*	73	*–*
Median	76	*–*	74	*–*	75	*–*
Range	40	*–*	36	*–*	40	*–*
Sex						
Female	14	*38.9*	24	*66.7*	38	*52.8*
Male	22	*61.1*	12	*33.3*	34	*47.2*
Prioritised ethnicity						
NZ European	32	*88.9*	34	*94.4*	66	*91.7*
Māori	2	*5.6*	2	*5.6*	4	*5.6*
European Other	1	*2.8*	0	*0.0*	1	*1.4*
Not Stated	1	*2.8*	0	*0.0*	1	*1.4*
Cancer type						
New primary diagnosis of colon cancer	27	*75.0*	26	*72.2*	52	*72.2*
Metastatic relapse of known colon cancer	3	*8.3*	1	*2.8*	4	*5.6*
New primary diagnosis of rectal cancer	4	*11.1*	9	*25.0*	13	*18.1*
Metastatic relapse of known rectal cancer	2	*5.6*	0	*0.0*	2	*2.8*
TNM stage grouping						
One	4	*11.4*	2	*6.9*	10	*13.9*
Two	14	*40.0*	9	*31.0*	24	*33.3*
Three	10	*28.6*	14	*48.3*	26	*36.1*
Four	7	*20.0*	4	*11.4*	12	*16.7*
Tumour grade						
Well-differentiated	0	*0.0*	0	*0.0*	0	*0.0*
Moderately differentiated	26	*74.3*	24	*82.8*	57	*79.2*
Poorly differentiated	5	*14.3*	4	*13.8*	9	*12.5*
Undifferentiated	0	*0.0*	0	*0.0*	0	*0.0*
Mucinous	4	*11.4*	1	*3.4*	6	*8.3*
Surgery for Primary Tumour	32	*88.9*	36	*100*	68	*94,4*
Elective/Planned	28	*87.5*	29	*80.6*	57	*83.8*
Acute - Emergency	4	*12.5*	5	*13.9*	9	*13.2*
Acute – Non-Emergency	0	*0*	2	*5.6*	2	*2.9*

Overall, participants had high levels of comorbidities that influenced their health and ability to carry out daily life activities. However, the intervention group patients were healthier than those in the observation group among most measured variables. Thus, fewer intervention patients were eligible for CMA (*n* = 29) than were deemed eligible in the observation group (*n* = 35) (Table 2).

**Table 2:** Level of comorbidity (total cohort)

	Observation		Intervention		Total	
	#	%	#	%	#	%
Eligible for CMA	35	*97.2*	29	*80.6*	64	*88.9*
Medication/polypharmacy						
3+ Medications	29	*80.6*	21	*58.3*	50	*69.4*
C, D or X Medication interaction	23	*63.9*	19	*52.8*	42	*58.3*
Hospital Admissions in the last 12 months						
None	20	*55.6*	28	*77.8*	48	*66.7*
One	9	*25.0*	4	*11.1*	13	*18.1*
Two or more	7	*19.4*	3	*8.3*	10	*13.9*
Health impact on Activities of Daily Life (Four most prevalent)						
Gets short of breath walking on flat surfaces	8	*22.2*	6	*16.7*	14	*19.4*
Needs to stay in bed or a chair most of the day	11	*30.6*	1	*2.8*	12	*16.7*
Has difficulty taking long walks	10	*27.8*	1	*2.8*	11	*15.3*
One or more falls or near falls in past 6 months	5	*13.9*	4	*11.1*	9	*12.5*
Health impact on Quality of Life						
Ability to work or do other daily activities	14	*38.9*	5	*13.9*	19	*26.4*
Ability to do hobbies	12	*33.3*	5	*13.9*	17	*23.6*
Social life	13	*36.1*	3	*8.3*	16	*22.2*
Family life	10	*27.8*	2	*5.6*	12	*16.7*

### The CMA and resultant management

Of the 29 intervention patients eligible for CMA, the referral was declined by eight patients. Thus, the CMA was delivered to 21 intervention group patients, most commonly post-operatively. Table 3 shows data for those 21 patients. The three health domains most commonly actively managed through CMA were cognition, memory, and anaemia. Cognitive testing was completed for 23.8% (*n* = 5) of the intervention group with cognitive deficits identified in all patients who were tested. Five patients had new diagnoses made; four of these patients had one new diagnosis made and one patient was diagnosed with three new conditions. On-going management was recommended for two (9.5%) patients (Table 3).

**Table 3:** Management made during CMA (CMA patients only, *n* = 21)

	Intervention	
	#	%
Health domains actively managed within CMA		
Anaemia	2	9.5
Nutrition	1	4.8
Plan in response to an abnormal response test	1	4.8
Bladder	1	4.8
Pain	1	4.8
Bowels	1	4.8
Postural hypotension	1	4.8
Renal	1	4.8
Gait or imbalance	1	4.8
Memory	4	19.0
On-going geriatrician management	2	9.5
Cognitive testing	5	23.8
Interventions occurring as a result of CMA		
Medications altered	1	4.8
Medications stopped	1	4.8
Medications started	2	9.5
One-off medication prescribed	1	4.8
Investigations ordered	4	19.0
Referrals made	2	9.5
Diagnoses made or removed	5	23.8

### Effect of CMA on patient outcomes

Outcome measures were able to be collected on all patients. Table 4 outlines the key outcomes of the study. Of the patients who received chemotherapy, no patients in the observation group completed chemotherapy as planned, whereas 6/11 (55%) of intervention patients completed chemotherapy without modification. There were a slightly higher proportion of unplanned hospitalisations in the intervention group (5/29) compared to the observation group (2/35) but little difference in terms of adverse events or attendance at an emergency department.

**Table 4:** Key outcome measures (total cohort)

Key Outcomes	Observation	Intervention
% referred to medical oncology	49% (17/35)	59% (17/29)
% received chemotherapy	40% (14/35)	38% (11/29)
Patient competed chemotherapy as planned	0% (0/14)	55% (6/11)
Patient had adverse event (grade 3/4)	29% (10/35)	28% (8/29)
Unplanned hospitalisation	6% (2/35)	17% (5/29)
Emergency clinic attendance	23% (8/35)	24% (7/29)

### Feasibility of comorbidity screening tools and acceptability of CMA

The self-reported comorbidity screening tool was completed by all patients and took on average 15 min to complete (range 5–40 min). All 13 patients interviewed indicated that our research requests were reasonable, including screening tool completion. However, the geriatricians suggested tightening the screening criteria triggering referral to their service. While a number of patients they saw were – on paper – comorbid, these comorbidities either did not impact greatly or were unable to be addressed.

The six CMA patients interviewed thought the CMA assessment was worthwhile, and reported useful outcomes, such as having a thorough medical exam, a decrease in prescribed medications or that somebody within the health system took an active interest in them. As discussed *“I just felt somebody was taking an interest in me, and … being caring, which I think that caring touch is most important in my life at the moment”.* However, the use of geriatricians had an unintended consequence with a number of the patients stating that a geriatrician consultation made them feel unduly elderly.

The geriatricians suggested that delivering the CMA pre-operatively may better alter cancer treatment and outcomes for comorbid patients. Each questioned their ability to initiate changes to the patients’ care plan, with recommendations often made to the patients’ primary care practitioner instead. They did, however, diagnose unrecognised cognitive impairment and intervene on a number of patients and both saw value in continuing to better manage comorbidity in cancer patients of all ages, with one stating *‘I … think it is an important area to be working within’*. Cancer nurse coordinators also saw value in better managing comorbidity and were willing to be more involved in the future.

### Feasibility of a larger-scale study

We anticipated that there may be lower enthusiasm for health-services style research than for studies of novel medications or devices that may be perceived to have a greater potential for health gain. We took several steps to mitigate this. We undertook workshops in advance of opening the study, and held meetings with key study and clinical staff in advance and at regular intervals during the study. We presented at clinical meetings, identified a local clinical champion to support the study, and each study site was reimbursed for research nurse and geriatrician administration time. Additionally, the criteria that triggered the invitation to CMA, the CMA intervention and CMA processes were developed with the geriatricians involved, and procedures established to inform each patient’s primary care practitioner of the CMA and its outcomes. Furthermore, we carried out two additional strategies after the study had commenced, specifically to maximise patient recruitment. Firstly, we amended the timing of recruitment from strictly pre-operatively to one that allowed patients to be enrolled post-operatively. This change was made on feedback from key study staff that patients were too overwhelmed in the initial phase of their cancer journey to participate in research. Secondly, we lengthened the duration of the study recruitment phase from 9 months to 12 months. Even after enacting these strategies we recruited fewer patients that we had planned for; 72 patients were enrolled, rather than the planned 124 patients.

The qualitative phase suggested that the study processes were acceptable with all of the 13 patients interviewed reporting that the research requests were reasonable. Additionally, the data for proposed outcome measures was feasible to collect and the key outcome measure (proportion completing chemotherapy as planned) appears to be a feasible outcome to measure. This suggests that larger studies of this intervention are possible. Thus despite the recruitment challenges encountered, the study team determined that we should proceed to a larger scale study, albeit one with some modification.

## Discussion

The aim of this pilot study was to assess the feasibility and acceptability of comprehensive medical assessment in comorbid patients of all ages with colorectal cancer, and the feasibility of conducting a large multi-centre study in NZ. Although the results of this study can only be considered indicative, the higher rate of chemotherapy completion in the intervention group is promising and feedback from patients and staff was largely positive, indicating acceptability of the approach. The information generated by this study has provided a solid foundation of learnings on which to further develop the coordinated approach to management of comorbid cancer patients proposed.

Screening for and active management of comorbidity soon after cancer diagnosis shows promise in positively altering cancer outcomes, such as improved surgical outcomes [26, 30], reduced risk of chemotherapy toxicity, and increased likelihood of completing chemotherapy as planned [23–26, 29, 39]. Similarly, we found that a higher proportion of patients undergoing our intervention completed chemotherapy without modification than those who did not (6/11 and 0/14 respectively). Although, as a pilot study, our patient numbers were small, this finding indicates some potential in this approach. Interpretation of the results is complicated by the fact that the intervention group was healthier than the observation group in most variables measured at baseline. Due to informal feedback, we suspect that research nurses were less inclined to approach sicker patients during the intervention phase, because they felt those patients may be already over-burdened. A randomised controlled trial (RCT) would reduce the variability observed between the patient groups. However, the effectiveness of service interventions are difficult to evaluate using an individual-level RCT framework due to potential contamination between control and intervention groups [40]. A cluster RCT would avoid these issues, but there may be scientific and practical problems with implementing such a study in NZ - primarily that there may be too few cancer centres in NZ to provide sufficient numbers of clusters. Other approaches such as a step-wedge trial may be useful but have limitations including additional data burden and complexity of analysis [40]. A non-randomised evaluation using a before and after approach, with mechanisms in place to ensure balanced invitation processes, may still be the best way to continue this work within the NZ context.

In order to progress to a larger-scale study, several changes are required to the study tools and processes. First, a screening tool with greater specificity is required. Although we found high levels of comorbidity in the study population (consistent with published literature, [7, 39]), it does appear that the threshold which triggered the referral to CMA was set too low within our study; both geriatricians indicated that the patients referred to them had few comorbidities that were amenable to intervention. Ideally, a screening tool should have not only high sensitivity but also high specificity to identify those comorbid conditions which are amenable to intervention, and so minimise patients being referred unnecessarily [41, 42]. To date, no one gold standard tool has been developed for the identification of important comorbidity [23, 24, 41, 42], although the G-8 tool has promise for correctly identifying older cancer patients who might benefit from a CGA [43]. The G-8 tool includes questions on nutritional status/food intake, weight loss, body mass index, motor skills, psychological status, number of medications, and patient-perception of health, along with age > 80 years as criteria [43]. The G-8 tool is designed to be completed by trained nurses, whereas our study utilised a mix of patient self-report and clinical note review by nurses to determine eligibility for CMA. However, a self-administered screening tool consisting of 14 measures has also been shown to be feasible to administer in the hospital setting [42], and helpful in selecting patients who will most benefit from a more formal comorbidity management process [42, 44]. The design of an effective screening tool that correctly identifies which cancer patients will most benefit from additional management of their comorbid conditions prior to cancer treatment is vital to any future approach in this area.

Secondly, the CMA itself requires refinement. While the findings of our qualitative work indicate acceptability of the CMA intervention, the geriatricians each questioned their mandate to change patient management. Geriatricians typically receive referrals with a presenting problem clearly identified, whereas, in this study, referral occurred if patients met certain study criteria. A number of management recommendations were made to the patients’ primary care team within clinic letters rather than enacted within the CMA itself. It is possible that the CMA not be delivered by geriatricians. A revised CMA using a different health professional - such as cancer specialist nursing and/or the wider oncology or surgical teams [42] - to act on the results of a comorbidity screen is plausible. If the geriatrician-led CMA model continues with a more select group of patients, consideration should be given to providing patients with more information about the intervention at point-of-referral and, conversely, providing more information about the patients to the geriatricians. Further engagement with other health professionals, including primary care practitioners [45], will also be integral to future refinement of the study.

Thirdly, findings from this study suggest that the CMA should be introduced early in the cancer treatment pathway. The recruitment focus in this study was changed from soon after diagnosis to post-operatively on feedback from research nurses indicating that they felt that patients would be unable or unwilling to participate in research at this early point in their cancer journey. While it is plausible that those patients with the greatest and most complex health needs - who might benefit most from a comprehensive comorbidity-management intervention - may be least likely to participate in research, information from the qualitative phase of this feasibility study suggests that earlier patient recruitment and thus earlier CMA for those eligible would have been acceptable. All 13 patients interviewed – including four patients enrolled pre-operatively - indicated that that research requests were reasonable, and the geriatricians stated that they believed a pre-operative CMA would have been more useful. As emphasised by a cancer nurse coordinator ‘*patients will be overwhelmed soon after diagnosis but this should not stop us enacting things that will improve their care’.* Previous studies have identified patients for CMA once they had already been selected for a treatment such as surgery or chemotherapy, but before treatment was commenced [29]. Pre-operative CMA has shown to be useful to identify and better manage cancer patients likely to experience post-operative complications, including delirium [42]. It is likely pre-operative intervention would have been equally useful in this study and should be considered in the future.

Finally, whatever screening tool and comorbidity management model are utilised in the future, it is important that they be well accepted and effectively used in practice; participatory research approaches with key hospital staff may best enable this [46–48]. Participatory research is compatible with current implementation science [49, 50], with increasing attention being given to researchers, end-users and decision-makers working together to design and subsequently evaluate health-care interventions [46, 51–53]. In addition to a participatory research approach, a combination of strategies such as those we enacted to gain buy-in to this health service intervention research will be critical to any future study that intends to transform clinical practice or modify a complex cancer treatment pathway. Process evaluation to better understand how and why the study succeeds along with economic evaluation of any future study should also occur (Table 5).

**Table 5:** Summary of planned alterations to improve the success of the study

Issue	Solution
Improved recruitment numbers	Investigate a service level intervention whereby individual patients are not recruited, i.e. a clustered roll out across multi-centres Enact the strategies used within this pilot study e.g. workshops and meetings at regular intervals, identify local clinical champions, but allow a longer lead-in time for each centre before they are recruited into the study Have clear accountability for recruitment numbers to the study team Increase the number of potentially eligible participants by including a wider range of cancer types Have three defined points at which patients are recruited 1) pre-elective surgery outpatient clinics as part of standard care, 2) at pre-operative MDM, and 3) at point or referral to medical oncology assessment, if not already recruited at point 1) or 2)
Pre-operative recruitment and CMA	Include pre-operative recruitment as a study criteria with allowance for point 3) above Develop processes so that the CMA intervention is delivered pre-operatively
Variability observed between the patient groups minimised	Ensure mechanisms are in place to ensure balanced invitation processes e.g. randomisation of services (using a cluster approach) and/or recruitment of consecutive patients over a defined period
Screening tool with greater specificity	Carry out a literature review and iterative design of a screening tool applicable for the NZ context
Intervention modified to better integrate into clinical pathways	Consider other models of intervention e.g. CMA carried out by cancer specialist nursing, surgical or oncology team with referrals as needed; CMA carried out and oncology team provided with results to take necessary action; primary care practitioner provided with results to take necessary action
Screening tool and intervention well accepted and used in practice	Provide patients with more information about the intervention at point-of-referral Enable participatory research approaches with key hospital staff including clinicians, nursing, service managers

The key strengths of this study are that - to our knowledge - no studies to date have investigated a comorbidity optimisation intervention for cancer patients of all ages. Additionally, this study utilised a mixed methodological approach involving key study staff and patients to assess on-going feasibility of the approach. This study also had several important limitations. First, small study numbers prevent reporting anything other than descriptive statistics. However, as this was a pilot study no formal hypothesis testing was planned. Rather, this study was designed to provide critical information to inform a larger study. Secondly, this study investigated colorectal cancer only and the findings are not directly generalisable to other cancers. Thirdly, the study is specific to the NZ health system. However, the key outcome finding that patients undergoing comprehensive management of their comorbid conditions appeared more likely to complete chemotherapy treatment as planned supports findings in previous comparable studies investigating a range of cancers in the United Kingdom [29] and United States of America [30].

## Conclusion

This pilot study showed promise in its key outcome measure and the modified model of care – geriatrician-led CMA – was acceptable to comorbid cancer patients. Importantly, the study has provided useful insight into how the study can be implemented in the future.

## Supplementary information

**Additional file 1:** CONSORT extension for pilot and feasibility trials checklist.

**Additional file 2:** CMA criteria.

## Data Availability

Data and study material are available on request to the corresponding author.

## References

[1] Sarfati D, Hill S, Blakely T, Robson B, Purdie G, Dennett E (2009). The effect of comorbidity on the use of adjuvant chemotherapy and survival from colon cancer: a retrospective cohort study. BMC Cancer.

[2] Munro AJ, Bentley AH (2004). Deprivation, comorbidity and survival in a cohort of patients with colorectal cancer. European journal of cancer care.

[3] Extermann M (2000). Measurement and impact of comorbidity in older cancer patients. Crit Rev Oncol Hematol.

[4] Extermann M (2000). Measuring comorbidity in older cancer patients. Eur J Cancer.

[5] Sarfati Diana, Gurney Jason, Lim Bee Teng, Bagheri Nasser, Simpson Andrew, Koea Jonathan, Dennett Elizabeth (2013). Identifying important comorbidity among cancer populations using administrative data: Prevalence and impact on survival. Asia-Pacific Journal of Clinical Oncology.

[6] Sarfati D, Gurney J, Stanley J (2014). Salmond C, Crampton P, Dennett E, et al. Cancer-specific administrative data-based comorbidity indices provided valid alternative to Charlson and NHI indices. J Clin Oncol.

[7] Sarfati D, Koczwara B, Jackson C (2016). The impact of comorbidity on cancer and its treatment. CA Cancer J Clin.

[8] Sogaard M, Thomsen RW, Bossen KS, Sorensen HT, Norgaard M (2013). The impact of comorbidity on cancer survival: a review. Clinical epidemiology.

[9] Institute of Medicine (2013). Delivering high-quality cancer care: charting a new course for a system in crisis.

[10] Sarfati D, Gurney J, Stanley J, Koea J (2014). A retrospective cohort study of patients with stomach and liver cancers: the impact of comorbidity and ethnicity on cancer care and outcomes. BMC Cancer.

[11] Boakye D, Rillmann B, Walter V, Jansen L, Hoffmeister M, Brenner H (2018). Impact of comorbidity and frailty on prognosis in colorectal cancer patients: a systematic review and meta-analysis. Cancer Treat Rev.

[12] Brewer N, Borman B, Sarfati D, Jeffreys M, Fleming ST, Cheng S (2011). Does comorbidity explain the ethnic inequalities in cervical cancer survival in New Zealand? A retrospective cohort study. BMC Cancer.

[13] Lee L, Cheung WY, Atkinson E, Krzyzanowska MK (2011). Impact of comorbidity on chemotherapy use and outcomes in solid tumors: a systematic review. J Clin Oncol.

[14] Chaudhary RK, Bhaduri D, Bhatia M, Hatti S, Ba R, Meva J (2013). Influence of comorbidity in cancer surgery on treatment decisions, postoperative course and oncological outcome. Asia Pac J Clin Oncol.

[15] Iachina M, Green A, Jakobsen E (2014). The direct and indirect impact of comorbidity on the survival of patients with non-small cell lung cancer: a combination of survival, staging and resection models with missing measurements in covariates. BMJ Open.

[16] Kaerlev L, Iachina M, Trosko O, Qvist N, Ljungdalh PM, Nørgård BM (2018). Colon cancer patients with a serious psychiatric disorder present with a more advanced cancer stage and receive less adjuvant chemotherapy - a Nationwide Danish cohort study. BMC Cancer.

[17] Seigneurin A, Delafosse P, Trétarre B, Woronoff AS, Velten M, Grosclaude P (2018). Are comorbidities associated with long-term survival of lung cancer? A population-based cohort study from French cancer registries. BMC Cancer.

[18] Tetsche MS, Dethlefsen C, Pedersen L, Sorensen HT, Norgaard M (2008). The impact of comorbidity and stage on ovarian cancer mortality: a nationwide Danish cohort study. BMC Cancer.

[19] Bradley CJ, Dahman B, Anscher M (2014). Prostate cancer treatment and survival: evidence for men with prevalent comorbid conditions. Med Care.

[20] Grann AF, Froslev T, Olesen AB, Schmidt H, Lash TL (2013). The impact of comorbidity and stage on prognosis of Danish melanoma patients, 1987-2009: a registry-based cohort study. Br J Cancer.

[21] Gross CP, McAvay GJ, Guo Z, Tinetti ME (2007). The impact of chronic illnesses on the use and effectiveness of adjuvant chemotherapy for colon cancer. Cancer..

[22] Jehn CF, Boning L, Kroning H, Pezzutto A, Luftner D (2014). Influence of comorbidity, age and performance status on treatment efficacy and safety of cetuximab plus irinotecan in irinotecan-refractory elderly patients with metastatic colorectal cancer. Eur J Cancer(Oxford, England : 1990).

[23] Puts MT, Hardt J, Monette J, Girre V, Springall E, Alibhai SM (2012). Use of geriatric assessment for older adults in the oncology setting: a systematic review. J Natl Cancer Inst.

[24] Puts MT, Santos B, Hardt J, Monette J, Girre V, Atenafu EG (2014). An update on a systematic review of the use of geriatric assessment for older adults in oncology. Ann Oncol.

[25] Wildiers H, Heeren P, Puts M, Topinkova E, Janssen-Heijnen ML, Extermann M (2014). International Society of Geriatric Oncology consensus on geriatric assessment in older patients with cancer. J Clin Oncol.

[26] Extermann M, Hurria A (2007). Comprehensive geriatric assessment for older patients with cancer. J Clin Oncol.

[27] Ramjaun A, Nassif MO, Krotneva S, Huang AR, Meguerditchian AN (2013). Improved targeting of cancer care for older patients: a systematic review of the utility of comprehensive geriatric assessment. J Geriatr Oncol.

[28] Yokom DW, Alibhai SMH, Sattar S, Krzyzanowska MK, Puts MTE (2018). Geriatric oncology screening tools for CGA-based interventions: results from a phase II study of geriatric assessment and management for older adults with cancer. J Geriatr Oncol.

[29] Kalsi T, Babic-Illman G, Ross P, Maisey N, Hughes S, Fields P (2015). The impact of comprehensive geriatric assessment interventions on tolerance to chemotherapy in older people. Br J Cancer.

[30] McCorkle R, Strumpf NE, Nuamah IF, Adler DC, Cooley ME, Jepson C (2000). A specialized home care intervention improves survival among older post-surgical cancer patients. J Am Geriatr Soc.

[31] Rao AV, Hsieh F, Feussner JR, Cohen HJ (2005). Geriatric evaluation and management units in the care of the frail elderly cancer patient. J Gerontol A Biol Sci Med Sci.

[32] Soejono C (2006). The role of comprehensive geriatric assessment (CGA) in the management of stage 3 hepatocellular carcinoma in the elderly. Crit Rev Oncol Hematol.

[33] Ministry of Health (2015). Content guide 2014/15: New Zealand health survey.

[34] Aaronson N. K., Ahmedzai S., Bergman B., Bullinger M., Cull A., Duez N. J., Filiberti A., Flechtner H., Fleishman S. B., Haes J. C. J. M. d., Kaasa S., Klee M., Osoba D., Razavi D., Rofe P. B., Schraub S., Sneeuw K., Sullivan M., Takeda F. (1993). The European Organization for Research and Treatment of Cancer QLQ-C30: A Quality-of-Life Instrument for Use in International Clinical Trials in Oncology. JNCI Journal of the National Cancer Institute.

[35] Small W, Pugh S, Wagner L, Kirshner J, Sidhu K, Bury M (2013). RTOG 0841: Two-Item Questionnaire Effectively Screens for Depression in Cancer Patients Receiving Radiation Therapy. Int J Radiat Oncol Biol Phys.

[36] Mitchell AJ (2008). Are one or two simple questions sufficient to detect depression in cancer and palliative care? A Bayesian meta-analysis. Br J Cancer.

[37] Wolters Kluwer Clinical Drug Information. Lexi-comp online user guide. 2012. Available from: http://www.wolterskluwercdi.com/lexicomp-online/. Accessed 22 May 2019.

[38] U.S.Department of Health and Human Service (2010). Common Terminology Criteria for Adverse Events (CTCAE) Version 4.03. National Cancer Institute.

[39] Freyer G, Geay JF, Touzet S, Provencal J, Weber B, Jacquin JP (2005). Comprehensive geriatric assessment predicts tolerance to chemotherapy and survival in elderly patients with advanced ovarian carcinoma: a GINECO study. Ann Oncol.

[40] Barratt H, Campbell M, Moore L, Zwarenstein M, Bower P, Raine R, Fitzpatrick R, Barratt H (2016). Essay 2. Randomised controlled trials of complex interventions and large-scale transformation of services. Challenges, solutions and future directions in the evaluation of service innovations in health care and public health. 4.

[41] Hamaker ME, Jonker JM, de Rooij SE, Vos AG, Smorenburg CH, van Munster BC (2012). Frailty screening methods for predicting outcome of a comprehensive geriatric assessment in elderly patients with cancer: a systematic review. The Lancet Oncology.

[42] Hernandez Torres C, Hsu T (2017). Comprehensive geriatric assessment in the older adult with Cancer: a review. Eur Urol Focus.

[43] Bellera CA, Rainfray M, Mathoulin-Pélissier S, Mertens C, Delva F, Fonck M (2012). Screening older cancer patients: first evaluation of the G-8 geriatric screening tool. Ann Oncol.

[44] Kalsi T, Babic-Illman G, Hughes S, Ross P, Fields P, Brodie H (2014). Validity and reliability of a comprehensive geriatric assessment screening questionnaire (CGA-GOLD) in older people with cancer. Age Ageing.

[45] Perfors IAA, May AM, Boeijen JA, de Wit NJ, van der Wall E, Helsper CW (2019). Involving the general practitioner during curative cancer treatment: a systematic review of health care interventions. BMJ Open.

[46] Meyer J (2000). Using qualitative methods in health related action research. Br Med J.

[47] Waterman H, Tillen D, Dickson R, de Koning K. Action Research: a systematic review and guidance for assessment: Health Technology Assessment [Internet]. 2001 22/05/2019; 5(23). Available from: http://www.hta.ac.uk/execsumm/summ523.shtml. 11785749

[48] Williamson G, Posser S (2002). Action research: politics, ethics and participation. J Adv Nurs.

[49] Holtrop JS, Rabin BA, Glasgow RE (2018). Dissemination and implementation science in primary care research and practice: contributions and opportunities. J Am Board Fam Med.

[50] Mitchell SA, Chambers DA (2017). Leveraging implementation science to improve Cancer care delivery and patient outcomes. J Oncol Pract.

[51] Boyd H, McKernon S, Mullin B, Old A. Improving healthcare through the use of co-design. New Zealand Medical Journal. 2012;125(1357):76-87. 22854362

[52] Eyles H, Jull A, Dobson R, Firestone R, Whittaker R, Te Morenga L (2016). Co-design of mhealth delivered interventions: a systematic review to assess key methods and processes. Curr Nutr Rep.

[53] Cargo M, Mercer SL (2008). The value and challenges of participatory research: strengthening its practice. Annu Rev Public Health.

