# Supplementary material for: Improving management of comorbidity in patients with colorectal cancer using comprehensive medical assessment: a pilot study
Source: BMC Cancer. 2020 Jan 20;20:50. doi: 10.1186/s12885-020-6526-z (PMC6971855; doi:10.1186/s12885-020-6526-z)
Supplement: Supplementary file 2 — Additional file 2: CMA criteria. [file 12885_2020_6526_MOESM2_ESM.docx]

| **Supplementary material: Red and yellow criteria to trigger referral to the CMA intervention** |  |
| --- | --- |
| Red Criteria: Any ONE triggers an invite and referral to CMA |  |
| 3 or more medications |  |
| Medication Interactions at levels C, D or X |  |
| Admitted to hospital 2 or more times in the last 12 months or does not know if has been admitted |  |
| On insulin, or oral medication for diabetes both controlled and not well-controlled |  |
| Heart Failure |  |
| Stroke other than TIAs |  |
| Peripheral Vascular Disease |  |
| Severe Liver disease - include cirrhosis, chronic hepatitis |  |
| Dementia, Parkinson’s, Multiple Sclerosis |  |
| Substance or alcohol dependence or abuse |  |
| Has been seen by specialist doctor or community mental health team for depression OR Major depression – currently active not well controlled |  |
| Bipolar Disorder |  |
| Has been told by doctor they have an anxiety disorder/ currently on medication, well controlled or active, not well controlled |  |
| Schizophrenia and other psychoses |  |
| Connective Tissue Disease other than osteoarthritis or other non-specific arthritis |  |
| Chronic kidney disease |  |
| Obesity |  |
| Patient reports shortness of breath walking on flat surfaces |  |
| Patient reports one or more falls, or near falls, in last 6 months |  |
| Patient reports significant memory problems or patient does not know |  |
| Patient reports episodes of confusion or patient does not know |  |
| Patient reports difficulties with ADL or patient needs assistance - ‘Quite a bit’ or ‘A lot’ – **except** driving, taking public transport or managing financial affairs |  |
| Patient reports trouble taking a short walk outside of house - ‘Quite a bit’ or ‘Very much’ |  |
| Patient reports needs to stay in bed or a chair during the day - ‘Quite a bit’ or ‘Very much’ |  |
| Patient reports needs help with eating, dressing, washing or toileting - ‘Quite a bit’ or ‘Very much’ |  |
| Patient reports limited in work or other daily activities in last week - ‘Quite a bit’ or ‘Very much’ |  |
| Patient reports limited in pursuing hobbies or other leisure activities in last week - ‘Quite a bit’ or ‘Very much’ |  |
| Patient reports short of breath in last week - ‘Quite a bit’ or ‘Very much’ |  |
| Patient reports felt tense in last week - ‘Quite a bit’ or ‘Very much’ |  |
| Patient reports worried in last week - ‘Quite a bit’ or ‘Very much’ |  |
| Patient reports difficulty remembering things in last week - ‘Quite a bit’ or ‘Very much’ |  |
| Yellow Criteria: Any TWO OR MORE triggers an invite and referral to CMA |  |
| Admitted to hospital once in the last 12 months (assess alongside reasons for hospitalisation, not including admissions likely to be related to cancer diagnosis e.g. anaemia or abdominal pain) |  |
| Patient reports needing someone to help them fill in the Patient Health Questionnaire |  |
| Diabetes |  |
| Hypothyroidism, other endocrine disorders |  |
| Hypertension |  |
| Previous heart attack or angina or coronary artery disease |  |
| Cardiac arrhythmia, valvular disease, previous pulmonary embolism |  |
| ‘Other’ heart disease or Other CV system disorder |  |
| Chronic bronchitis or emphysema |  |
| Asthma, other respiratory disease |  |
| Non – iron deficiency anaemia |  |
| Other haematologial disorder i.e. clotting disorder, thalassaemias, myelodysplasia |  |
| Liver disease, includes heamochromatosis, Wilsons disease – do not include cancers |  |
| Inflammatory bowel disease i.e. crohns, ulcerative colitis |  |
| Previous stroke or TIAs, epilepsy or other neuro conditions |  |
| Leukaemia, Lymphoma, solid tumour |  |
| **NOTE: if two of these are checked and are the only yellow criteria checked** **– this counts as one yellow criteria** Patient states:   - have been told by a doctor they have depression - currently on regular pills for depression   OR Depression, History noted not currently active OR currently active but controlled |  |
| History of anxiety disorder noted but not currently active |  |
| Any other long term health conditions identified by pt. or in medical notes, unless very minor |  |
| Patient reports poor vision that limits patient |  |
| Patient reports difficulties with Activities of Daily Living (specifically driving, taking public transport or managing financial affairs) - ‘Quite a bit’ or ‘A lot’ |  |
| **NOTE: if two of these are checked and are the only yellow criteria checked – this counts as one yellow criteria** Patient reports NO to ‘is there a friend , relative or neighbour who:   - can take care of you for a few days if needed? - you feel you can talk to about your suspected cancer or cancer? |  |
| Patient lives alone or in sheltered housing |  |
| Patient reports being a caregiver for someone who depends on them? |  |
| Patient reports trouble doing strenuous activities or taking a long walk – ‘very much’ |  |
| Patient reports difficulty concentrating in last week - ‘Quite a bit’ or ‘Very much’ |  |
| Patient reports feeling irritable in last week - ‘Quite a bit’ or ‘Very much’ |  |
| Patient reports feeling depressed in last week - ‘Quite a bit’ or ‘Very much’ |  |
